# Pearl Sac Gene Expression Profiles Associated With Pearl Attributes in the Silver-Lip Pearl Oyster, *Pinctada maxima*

**DOI:** 10.3389/fgene.2020.597459

**Published:** 2021-01-08

**Authors:** Carmel McDougall, Felipe Aguilera, Ali Shokoohmand, Patrick Moase, Bernard M. Degnan

**Affiliations:** ^1^Centre for Marine Science, School of Biological Sciences, The University of Queensland, St. Lucia, QLD, Australia; ^2^Australian Rivers Institute, Griffith University, Nathan, QLD, Australia; ^3^Clipper Pearls and Autore Pearling, Broome, WA, Australia

**Keywords:** *Pinctada maxima*, pearl, pearl quality, nacre, biomineralization, CEL-Seq

## Abstract

Pearls are highly prized biomineralized gemstones produced by molluscs. The appearance and mineralogy of cultured pearls can vary markedly, greatly affecting their commercial value. To begin to understand the role of pearl sacs—organs that form in host oysters from explanted mantle tissues that surround and synthesize pearls—we undertook transcriptomic analyses to identify genes that are differentially expressed in sacs producing pearls with different surface and structural characteristics. Our results indicate that gene expression profiles correlate with different pearl defects, suggesting that gene regulation in the pearl sac contributes to pearl appearance and quality. For instance, pearl sacs that produced pearls with surface non-lustrous calcification significantly down-regulate genes associated with cilia and microtubule function compared to pearl sacs giving rise to lustrous pearls. These results suggest that gene expression profiling can advance our understanding of processes that control biomineralization, which may be of direct value to the pearl industry, particularly in relation to defects that result in low value pearls.

## Introduction

Pearls are stunning and structurally complex biominerals fabricated by a wide range of molluscs (Strack, 2006; Southgate and Lucas, 2008; McDougall et al., 2013b). Some species produce pearls composed of nacre (mother-of-pearl), and many of these species have been used for the production of cultured pearls, resulting in a valuable aquaculture industry (Australian Bureau of Agricultural and Resource Economics and Sciences [ABARES], 2018).

Cultured saltwater pearl production involves two oysters: a donor and a host. Small pieces of the mantle—the organ responsible for shell formation in molluscs—are excised from the donor oyster and surgically inserted into the gonad of the host, along with a spherical bead known as the nucleus (Taylor and Strack, 2008). Over time, the explanted mantle grows around the nucleus to form a continuous epithelial layer, the pearl sac (Taylor and Strack, 2008; McDougall et al., 2013b). The pearl sac first secretes an organic layer onto the surface of the nucleus (Taylor and Strack, 2008). This is followed by the deposition of successive layers of calcium carbonate, first prismatic and then nacreous, although a large degree of variation can be observed in individual pearls (Cuif et al., 2008, 2011; Mariom et al., 2019). This structural layering is similar to that observed within the pearl oyster shell that also consists of three layers; an outer organic-rich layer (the periostracum), a middle prismatic layer of calcite, and an inner nacreous layer of aragonite. These similarities have led to the generalized assertion that pearls are essentially inverted shells (Farn, 1986; Taylor and Strack, 2008).

The formation of pearls and shells by similar processes is evident at the molecular level. The proteinaceous component of adult pearl oyster (*Pinctada*) shells is complex, comprising over 80 individual shell matrix proteins (SMPs), many of which are specific to particular shell layers (Joubert et al., 2010; Marie et al., 2012; Liu et al., 2015). Gene expression analysis of pearl sacs has revealed that pearl formation also involves many of these previously identified SMPs (Wang et al., 2009; Inoue et al., 2010; McGinty et al., 2012; Zhan et al., 2013; Le Luyer et al., 2019); however, pearl sac specific isoforms of known biomineralization genes have also been reported (Kinoshita et al., 2011). Temporal transcriptomic analysis has further revealed that SMPs associated with the prismatic shell layer are up-regulated in the early stages of pearl formation, whereas those associated with the nacreous shell layer are up-regulated later (Mariom et al., 2019), suggesting that the molecular process of pearl formation largely recapitulates that observed in the shell.

**FIGURE 1:**
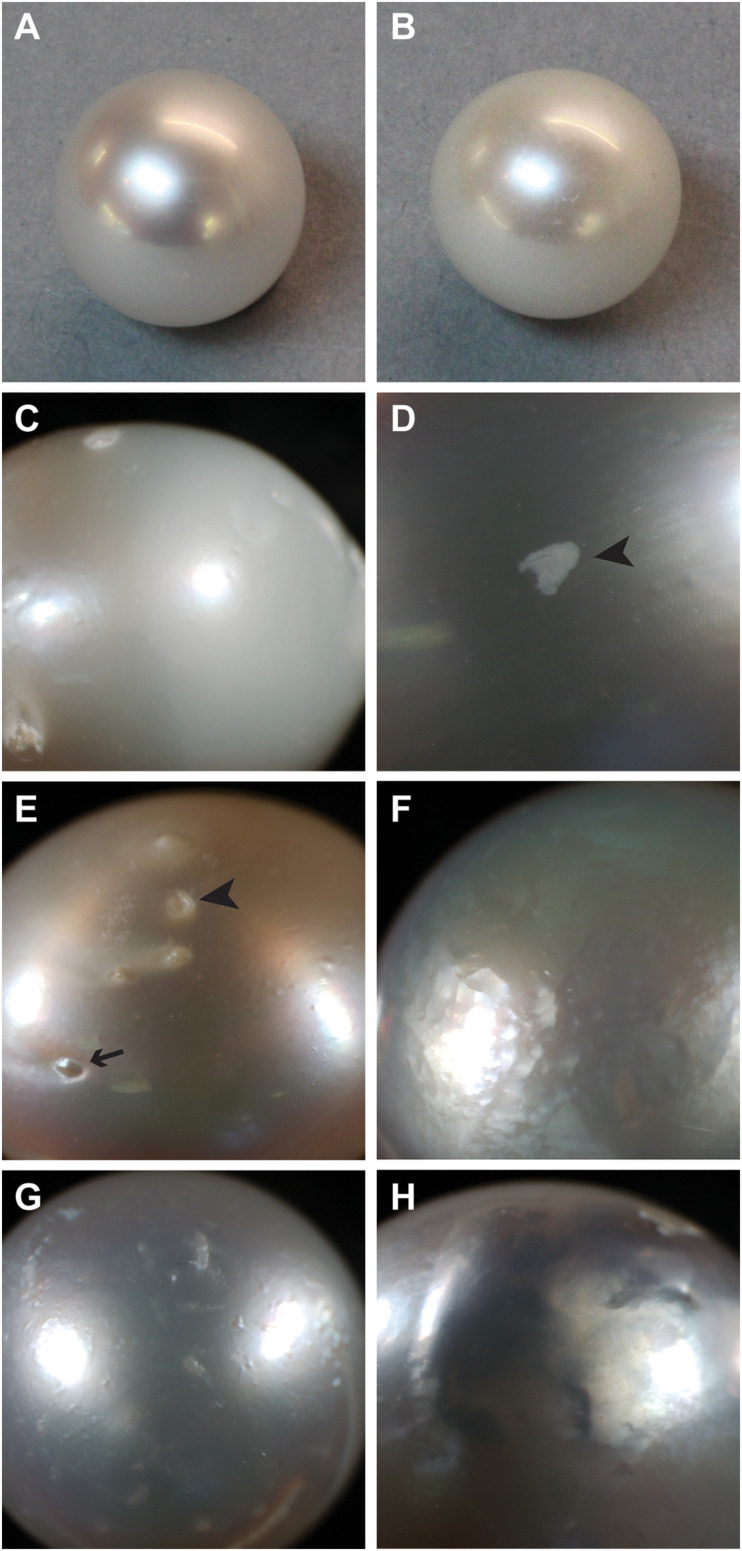
Examples of surface characteristics of pearls. **(A)** High luster, note sharp reflection. **(B)** Low luster. **(C)** Major calcification, note opaque appearance on right half of pearl. **(D)** Localized calcification (arrowhead). **(E)** Spots, both raised (arrow) and depressed (arrowhead). **(F)** Hammer. **(G,H)** Underskin.

From a commercial perspective, the ideal pearl is round, highly lustrous (shiny), of a pleasing color, and has an unblemished surface (Southgate and Lucas, 2008). However, many cultured pearls do not have these characteristics. For pearl oysters, seeding experiments have provided some insights into the underlying causes of some of the undesirable characteristics commonly found in pearls, and have indicated ways in which they might be avoided. For example, pearl shape is influenced by the skill of the grafting technician, and improvements can be made by modification of seeding techniques (Ky et al., 2015). Likewise, there is some evidence that luster (Figures 1A,B) and color can be improved by careful selection of donor oysters (Ky et al., 2014, 2019; Zhifeng et al., 2014; McDougall et al., 2016a; Blay et al., 2017). Surface blemishes, or defects, continue to be a problem for the pearl industry, despite some research indicating that these defects can, in some cases, be associated with particular host characteristics such as overall growth rate (McDougall et al., 2016a), or nacre deposition rate (Blay et al., 2014).

Given that molecular processes within the pearl sac ultimately regulate pearl formation, several studies have investigated potential links between pearl quality and gene expression. Inoue et al. (2010) assessed the expression levels of six candidate SMPs in pearl sacs that produced low- or high-quality pearls, assessed by the proportion of the pearl surface that displayed no defects. One gene (*msi31*) was found to be consistently up-regulated in high quality pearls. In a similar approach, Blay et al. (2018) determined the expression levels of eight candidate genes, representing both prismatic and nacreous SMPs, in pearl sacs. They found that three of the prismatic SMPs were up-regulated in sacs that produced pearls with low surface quality, whereas PIF (characterized as a nacreous SMP) was up-regulated in pearls with high surface quality (in this case, pearls with over 10 pits, bumps, or scratches were determined to have low surface quality). While these studies demonstrate that correlative differences can be observed between gene expression and pearl quality, differing expression of SMPs is likely to be a result of abnormal upstream processes, rather than the root cause.

In a recent study, Le Luyer et al. (2019) performed whole transcriptome analysis to compare gene expression between pearl sacs producing pearls of differing quality. The study revealed few genes (16) that were up-regulated in high quality pearls, compared to 246 up-regulated in low quality pearls. Again, an association between prismatic layer SMPs and poor pearl quality was detected. Although the study was not able to determine specific mechanisms that control pearl quality, the results did suggest a potential role for transposable elements, and potentially alternative splicing of biomineralization genes, on pearl characteristics.

The different ways in which pearl “quality” is determined may explain why the causes of poor pearl quality remain elusive. There are a number of different kinds of defects (McDougall et al., 2016a), and each type may have a different underlying cause. “Luster” describes the “shine” of the pearl, with high-luster pearls having a mirror-like reflectance and low-luster pearls appearing dull and are deemed low quality. Luster is ultimately determined by the thickness of the brick-like nacre tablets, with particular thicknesses inducing a phase-shift in reflected light that produces an iridescence effect (Simkiss and Wada, 1980). Poor luster can also be caused by a defect known within the industry as “calcification.” The term “calcification” is clearly a misnomer, as the entire pearl is clearly calcified, however, within the pearling industry, the term refers to the presence of white or opaque (non-lustrous) areas on the pearl surface (Figures 1C,D). In freshwater pearls, a similar defect is caused by the deposition of vaterite rather than aragonite (Ma and Lee, 2006; Bourrat et al., 2012); however, it is not known how this shift is mediated. Other pearls possess “spots” on their external surface, these can be either raised or depressed, and can often be associated with localized calcification (Figure 1E). In some cases, areas of the pearl surface have a golf ball-like appearance, known as “hammer” (Figure 1F), and in other cases, the pearl surface is bumpy or wrinkly, a condition known as “underskin” (Figures 1G,H). It is unknown whether any of these defects have similar underlying causes, and therefore the pooling of pearls with these qualities into a single “low quality” category possibly leads to low power for the detection of the underlying causes of these disparate defects.

Here, we utilize a low-input RNA-Seq method (CEL-Seq2) to evaluate gene expression in 28 individual pearl sacs from *Pinctada maxima*. The method was originally derived for gene expression analysis within single cells (Hashimshony et al., 2012, 2016), but has also been applied to multi-celled samples such as individual embryos and larvae (Anavy et al., 2014; Levin et al., 2016; Say and Degnan, 2020). Analysis of genes that are differentially expressed between these pearl sacs reveals that each investigated character or defect is associated with a distinct molecular signature, and, therefore, that these defects likely have different underlying causes. We predict that further investigation of the mechanistic causes of these particular defects will not only point the pearling industry toward possible methods for their prevention, but will also reveal fundamental principles about the biomineralization process that may be applicable across other biocalcifying taxa.

## Materials and Methods

### Transcriptome Sequencing

*Pinctada maxima* adult mantle, juvenile mantle, and pearl sac tissues (six individuals per tissue) were provided by Clipper Pearls, Broome, Western Australia. Tissues were dissected, immediately placed in RNAlater (Sigma–Aldrich), and stored at 4°C overnight before transportation and long-term storage at −20°C. Sampled mantle tissue consisted of all mantle zones, i.e., both edge and pallial. Pearl sacs were initially dissected along with surrounding gonad tissue, and were further dissected to isolate the pearl sac epithelium away from other tissues after storage in RNAlater. RNA extractions were performed separately for each individual mantle or pearl sac sample. Extractions were performed using 1 mL of TRI Reagent (Sigma–Aldrich) as per the manufacturer’s instructions, using 1-bromo-3-chloropropane for phase separation, and 0.25 mL of isopropanol and 0.25 mL of high salt precipitation solution (0.8 M sodium citrate and 1.2 M sodium chloride) for precipitation. RNA from each sample was pooled in equimolar amounts for each sample type (adult mantle, juvenile mantle, and pearl sac) and quality was checked on a Bioanalyzer (Agilent). RNA was sent to Macrogen (Seoul, Korea) for library preparation using a TruSeq Stranded mRNA Sample Prep Kit (Illumina) and sequencing on a HiSeq2000 to generate between 60 and 70 million 100 bp paired-end reads per library. A transcriptome assembly was performed using reads from all three libraries (adult mantle, juvenile mantle, and pearl sac) using Trinity v. 2014-04-13, with quality trimming via Trimmomatic and normalization of reads. Resulting transcripts were annotated using Trinotate pipeline 3.1.1^1^ (Bryant et al., 2017) via similarity searching against Swissprot by BLAST (Altschul et al., 1990), Pfam (Finn et al., 2016) by hmmscan (Finn et al., 2011), and by association with Gene Ontology terms (Ashburner et al., 2000).

### Pearl Sac Sampling

Sampling was conducted during standard harvesting operations of a cohort of pearls (24 months post seeding; originally seeded within a 2-day period by a single technician) by Clipper Pearls Pty Ltd., Broome, Western Australia. Harvesting operations were observed and pearls with varying qualities identified. These pearls were extracted, individually bagged, numbered, and graded by a single expert pearl grader at Autore Pearls Pty Ltd., utilizing a modification of the Autore pearl grading and classification system known as the Autore Five S’s^TM^ South Sea Pearl Classification Guide (trademark and copyright held by Autore Pearls Pty Ltd.) (Pearlautore International Pty Ltd,, 2006; McDougall et al., 2016b). Host characteristics including shell dorso-ventral height, anterior–posterior width, and sex at harvest (either male, female, or non-reproductive) were recorded at time of harvest. Pearl weight was calculated as the final weight of the pearl in momme (1 momme = 3.75 g), minus the average weight of the inserted nucleus. After pearls were harvested, a clean nucleus was inserted into the pearl sac, which was then dissected from the animal and stored in RNAlater^TM^ (Ambion) overnight at 4°C before long-term storage at −20°C.

### Gene Expression Analysis

For extraction of pearl sac epithelia, samples were placed in a petri dish containing RNAlater^TM^ and dissected open to reveal the embedded nucleus. The nucleus was removed, and surrounding pearl sac tissue peeled away from the surrounding tissue using fine forceps. Any adhering non-epithelial tissue (displaying distinct fluffy texture) was removed before the pearl sac tissue was placed into TRI Reagent^®^ (Sigma–Aldrich). RNA extractions were performed according to the manufacturer’s instructions.

Individual sequencing of pearl sac transcriptomes was performed using the CEL-Seq2 protocol (Hashimshony et al., 2016), which utilizes early sample barcoding, 3’ end-tagging, and the inclusion of 6 nt unique molecule identifiers (UMIs) to generate high-sensitivity transcriptomes from low input starting material. 25 ng RNA and 0.5 μl ERCC spike-in (1:10,000 dilution) were added to the initial RNA/primer/ERCC/dNTP mix for each sample. Paired-end sequencing was performed on a HiSeq 2500 (rapid run mode), with a 15 bp read 1 and a 55 bp read 2. Transcript counts were generated using the CEL-Seq2 pipeline (Hashimshony et al., 2016), modified to accommodate a 55 bp read 2, to use the –norc and –a commands during BOWTIE mapping, and to perform counting using a “fake”.gtf file. This was generated using faSize and the following command: cat P_maxima_transcriptome_Sizes.fa | awk ‘{print $1“\tPinctada\texon\t1\t“$2”\t\.\t\ + \t.\tgene_id \““$1”\””}’ > Pinctada_transcriptome_Fake.gtf. UMI counts were converted to transcript numbers following the binomial method outlined in previous studies (Grün et al., 2014). Transcripts with very low counts (less than 30 reads across all 28 samples after transformation) were removed from the dataset entirely. Transcript isoforms with very similar counts across all samples were collapsed using the “collapseRows” and “connectivityBasedCollapsing” function within the WGCNA program in R.

Differential gene expression analysis was performed for each pearl attribute (luster, weight, spots, underskin, and calcification) using DESeq2 (v 1.16.1) (Love et al., 2014) using an adjusted *p*-value cut-off of 0.05. For the analysis of luster, the two pearls exhibiting “B” grade luster were excluded from the analysis. Transcript counts (normalized using blind variance stabilizing transformation in DESeq2) were used to generate heatmaps for visualization of differentially expressed genes using the packages pheatmap version 1.0.12 (Kolde, 2012) and RColorBrewer version 1.1-2 (Neuwirth, 2011) in R version 3.5.1 (R Core Team,, 2014). Expression was scaled by row z-scores for visualization. Analysis for functional over-representation within differentially expressed transcripts was performed using hypergeometric tests of “biological process” gene ontology categories within the BiNGO plugin (Maere et al., 2005) of Cytoscape (Shannon et al., 2003), along with the Trinotate annotation of the *P. maxima* transcriptome as a reference and a *p*-value (Benjamini–Hochberg FDR correction) cut-off of 0.01.

Differentially expressed transcripts were further investigated to determine whether they (i) were likely to encode SMPs based upon similarity to proteins that had previously been identified from molluscan shells, (ii) possibly had regulatory roles (specifically, whether they were likely to have transcription regulatory or signaling activity), or (iii) whether they had similarity to transcripts that had been associated with pearl quality in a previous study (Le Luyer et al., 2019). Similarity to SMPs was ascertained by performing BLASTP searches against an in-house database of published proteins that had previously been identified from the shells of other mollusc species (Marie et al., 2010, 2011, 2012, 2013b, 2017; Bédouet et al., 2012; Mann et al., 2012, 2018; Pavat et al., 2012; Zhang et al., 2012; Mann and Jackson, 2014; Gao et al., 2015; Liao et al., 2015, 2019; Liu et al., 2015; Arivalagan et al., 2016; Upadhyay et al., 2016; Le Pabic et al., 2017; Shimizu et al., 2018), using an e-value cut-off of 1e^–10^. Reciprocal BLAST searches were then performed against the parent taxon of the top BLAST hit in NCBI to provide evidence for transcript homology. As many SMPs possess repetitive, low complexity domains (Sudo et al., 1997; Jackson et al., 2010; Marie et al., 2010; McDougall et al., 2013a, 2016b), BLASTP searches were conducted without filtering for low-complexity regions and without compositional adjustment. Potential transcription factor or signaling activity was ascertained by searching GO term annotations for GO:0003700 (DNA-binding transcription factor activity), or for the phrase “signal.” Finally, comparisons were made between the differentially expressed transcripts identified here and those identified in the study by Le Luyer et al. (2019). As the sequence data from the Le Luyer manuscript were not available at the time of writing, the top BLAST hits to the Le Luyer transcripts (Supplementary Table S2 in Le Luyer et al., 2019) were downloaded and used in reciprocal BLAST searches.

### Phylogenetic Analyses

To provide support to computational annotation, alignments of transcripts of interest and related sequences were performed and edited within AliView (Larsson, 2014). Maximum likelihood phylogenetic analyses were conducted using RAxML version 8.2.11 (Stamatakis, 2014), with automatic model selection and 100 rapid bootstrap inferences. Resulting phylogenetic trees were visualized in FigTree (Rambaut, 2006).

## Results and Discussion

### Transcriptome Sequencing and Assembly

To obtain a comprehensive transcriptome to facilitate investigation into *P. maxima* biomineralization, sequencing was performed for three libraries (adult mantle, juvenile mantle, and pearl sac; six individuals in each library) on an Illumina HiSeq 2000. Reads from all three libraries were used to construct a combined transcriptome assembly, consisting of 185,077 transcripts, with a contig N50 of 1740 bp. Raw sequences and assembled transcripts are publicly available under NCBI BioProject PRJNA636870.

### Characteristics of Selected Pearls

Standard pearl harvesting operations were observed and 28 pearls and their corresponding pearl sacs were selected for sampling based upon pearl appearance. The characteristics of the selected pearls are outlined in Table 1, and photographs of the pearls can be found in Supplementary Figure S1. Gene expression in each pearl sac was assessed using CEL-Seq2, with a resulting average sequencing depth of 6.4 million reads per sample (ranging from 461,492 to 14,789,883 reads), and an average mapping rate of 67% (ranging from 62 to 72%). Genes that were significantly differentially expressed in pearl sacs producing pearls with different characteristics were identified using DESeq2. Only two pearls were found that exhibited “hammer” on their surface, therefore this defect was not analyzed further.

**TABLE 1:** Characteristics of pearls selected for this study.

Pearl ID	Luster	Spots	Hammer	Underskin	Calcification	Pearl weight (momme)	Host sex at harvest
1	A	Yes	No	No	No	0.45	Male
2	C	Yes	No	No	No	0.35	Non-reproductive
3	C	Yes	No	No	No	0.25	Non-reproductive
4	C/D	Yes	No	Yes	Yes	0.30	Male
5	A	Yes	Yes	No	Yes	0.55	Male
6	A	No	No	No	No	0.50	Non-reproductive
7	A	No	No	No	No	0.70	Male
8	A	Yes	No	No	Yes	0.45	Non-reproductive
9	C	Yes	Yes	Yes	No	0.55	Male
10	A	Yes	No	No	No	0.30	Male
11	B	No	No	No	No	0.40	Non-reproductive
12	C	Yes	No	No	Yes	0.45	Non-reproductive
13	A	Yes	No	No	No	0.40	Non-reproductive
14	C	Yes	No	Yes	No	0.20	Male
15	C	Yes	No	No	No	0.30	Non-reproductive
16	A	Yes	No	No	No	0.50	Male
17	A	Yes	No	No	No	0.55	Male
18	C	Yes	No	No	Yes	0.65	Male
19	A	No	No	Yes	No	0.85	Non-reproductive
20	B	No	No	No	No	0.75	Non-reproductive
21	A	Yes	No	No	Yes	0.35	Non-reproductive
22	A	No	No	No	No	0.80	Male
23	C	Yes	No	No	Yes	0.15	Male
24	C	Yes	No	Yes	No	0.70	Male
25	A	Yes	No	No	No	0.45	Female
26	C	Yes	No	No	No	0.35	Male
27	A	Yes	No	No	No	0.70	Male
28	C/D	Yes	No	Yes	Yes	0.30	Non-reproductive

### Luster

**FIGURE 2:**
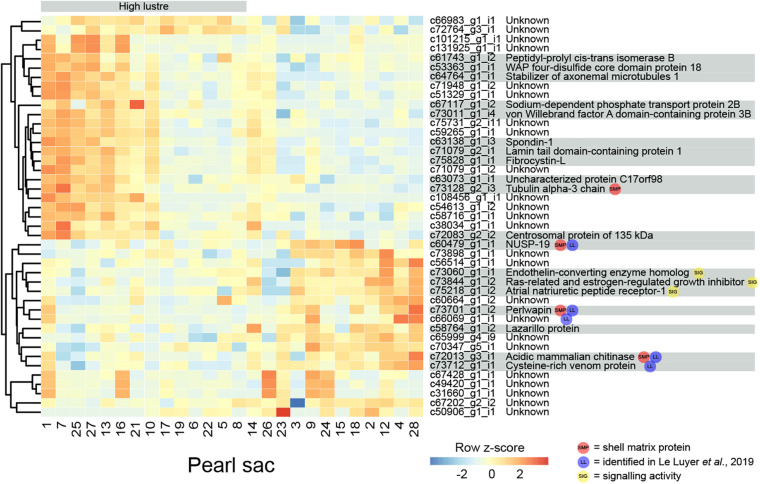
Heatmap of significantly differentially expressed transcripts associated with luster quality. The number at the bottom of each column corresponds to the pearl sac that produced the pearl displayed in Supplementary Figure S1. Transcripts are displayed as horizontal rows and are clustered by similarity of expression profiles, represented by the dendrogram to the left of the heatmap. Red indicates higher expression, and pearl sacs producing high luster pearls (A grade) are shown on the left of the heatmap, as indicated by the gray bar. Transcript annotations are indicated to the right of the heatmap.

Pearl luster is graded on a scale of A-D, with A grade pearls possessing greater luster. 43 transcripts are found to be significantly differentially expressed between pearl sacs producing high (A) and low (C or C/D) luster pearls, of which 19 have associated Swissprot annotations (Figure 2 and Supplementary Table S1). No specific biological process is over-represented in this dataset, most likely due to the low number of annotated transcripts.

Four differentially expressed transcripts are found to be highly similar to reported SMPs via reciprocal BLAST searches. Three are found to be down-regulated in high luster pearl sacs: c60479_g1_i1, which exhibits similarity to *Pinctada margaritifera* NUSP-19 (Marie et al., 2012); c72013_g3_i1, which is annotated as “acidic mammalian chitinase” and possesses similarity to SMPs found in nine bivalve species and in *Sepia officalis* cuttlebone; and c73701_g1_i2, which is annotated as perlwapin, a known SMP from abalone shells that inhibits calcium carbonate crystal growth *in vitro* (Treccani et al., 2006). The other transcript, c73128_g2_i3 (annotated as “tubulin alpha-3 chain”), is up-regulated in high luster pearl sacs. This alpha tubulin is almost identical at the amino acid level to proteins isolated from *Perna viridis* (99% similarity) and *Crassostrea gigas* (60% similarity) shells (Zhang et al., 2012; Liao et al., 2019). A number of intracellular proteins, including tubulins, have been detected within shells; however, it has been suggested that their presence is due to contamination of biominerals by cellular remains, i.e., that they are not true components of the organic matrix of shells (Marie et al., 2013a). Aside from putative SMPs, other differentially expressed genes exhibited similarity with genes that have been implicated in biomineralization in other species, for example, peptidyl-prolyl *cis-trans* isomerase (cyclophilin) (Amore and Davidson, 2006; Jackson et al., 2010) and spondin (Kinoshita et al., 2011; Funabara et al., 2014). Furthermore, five of the differentially expressed transcripts are detected in the pearl quality study by Le Luyer et al. (2019), including perlwapin, NUSP19, chitinase, “cysteine-rich venom protein” (c73712_g1_i1) and an unannotated transcript (c66069_g1_i1).

Three transcripts that are likely to have signaling functions and may be components of a genetic regulatory network that affects luster are co-expressed (Figure 2; c73060_g1_i1, annotated as endothelin-converting enzyme homolog; c73844_g1_i2, annotated as Ras-related and estrogen-regulated growth inhibitor; and c75218_g1_i2, annotated as atrial natriuretic peptide receptor-1). Although the functions of these signaling proteins are unstudied in molluscs and may differ from those in vertebrates (Grandchamp et al., 2019), the co-expression of these genes suggests that the regulatory interplay between these proteins (i.e., hydrolysis of atrial natriuretic peptide and regulation of Ras proteins by endothelin-converting enzyme (Foschi et al., 1997; Johnson et al., 1999) may be conserved.

### Calcification

**FIGURE 3:**
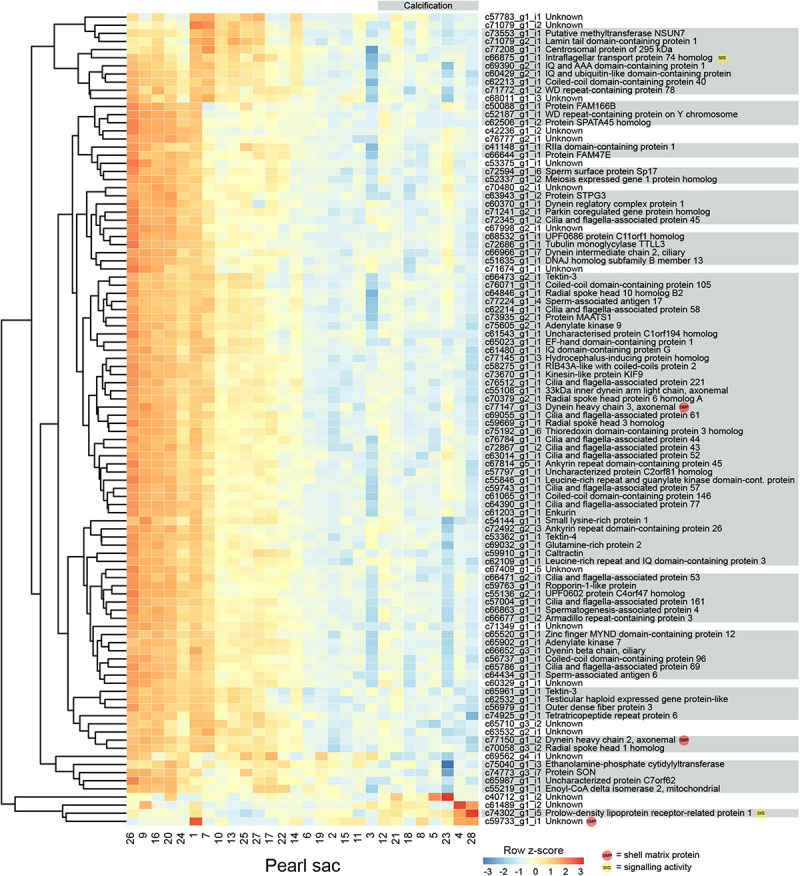
Heatmap of the 100 most significant calcification-associated differentially expressed transcripts. Transcripts are displayed as horizontal rows and are clustered by similarity of expression profiles. Red indicates higher expression, and pearl sacs producing pearls with calcification are shown on the right of the plot, as indicated by the gray bar. Transcript annotations are indicated on the right.

Eight pearls have some degree of calcification, and 315 transcripts are significantly differentially expressed between these pearls and those without the defect (Figure 3 displays the 100 most significant transcripts, see Supplementary Table S2 for the full list). 207 of these transcripts have Swissprot annotations, and 14 are similar to known SMPs. These include c72013_g3_i1, the transcript annotated as mammalian acidic chitinase that is also differentially expressed in the luster analysis, three unannotated transcripts, five transcripts with similarity to dynein proteins, and a number of other transcripts with similarity to intracellular proteins such as beta tubulin, pyruvate kinase, arginine kinase, and histone H3 (Supplementary Table S2). Three differentially expressed transcripts have similarity with transcripts associated with pearl quality in the study by Le Luyer et al. (2019), including two unannotated transcripts (c67849_g1_i1 and c72382_g1_i1), and a transcript annotated as metalloproteinase inhibitor 3 (c70381_g2_i1). A number of genes with potential signaling functions are differentially expressed (Supplementary Table S2), and one transcript (c58003_g1_i1) encoding the transcription factor forkhead box J1 (FoxJ1) is down-regulated in pearls with calcification (Supplementary Table S2 and Supplementary Figure S2). This result is congruent with the recent identification of *FoxJ1* as a candidate regulatory gene for expression of nacre-associated SMPs in the clam *Laternula elliptica* (Sleight et al., 2020).

Genes that are differentially expressed between sacs producing calcified and non-calcified pearls are enriched for genes involved in 96 biological processes (Supplementary Table S3, 10 most highly significant shown in Table 2), and many of these were associated with cilia or microtubule function, suggesting cellular cytoskeletal elements contribute to pearl formation and quality. Given that pearl sacs are located within the gonad of the host animal, we considered the possibility that this result could be due to sperm contamination from male gonads. This is unlikely to be the case, as the eight calcified pearls were obtained from four male hosts and four hosts that were reproductively inactive, and hosts producing non-calcified pearls had a similar sex distribution (Table 1).

**TABLE 2:** The 10 most highly enriched biological processes of transcripts differentially expressed between sacs producing calcified and uncalcified pearls.

Biological process	GO accession	Adjusted *P*-value
Cilium organization	0044782	3.56E-34
Cilium assembly	0060271	1.49E-31
Microtubule-based process	0007017	8.03E-30
Plasma membrane bounded cell projection assembly	0120031	5.52E-29
Cell projection assembly	0030031	7.38E-29
Microtubule-based movement	0007018	1.66E-26
Organelle assembly	0070925	2.12E-25
Cell projection organization	0030030	1.01E-23
Axoneme assembly	0035082	1.33E-19
Plasma membrane bounded cell projection organization	0120036	1.30E-18

The mantles of several different bivalves are known to be ciliated in different regions, including the larval and adult mantle of *Nodipecten nodosus* (Audino et al., 2015), and the inner mantle epithelium and folds of *Velesunio ambiguus* and *Hyridella depressa* (Colville and Lim, 2003). Ciliated mantle cells are also present in primary cell cultures from the bivalve clam *Paphia malabarica* (Dessai, 2012). In *P. margaritifera*, cilia are present in the epithelium of the inner fold, the periostracal groove, and the outer fold, and cells within the mantle pallial have “short protruding cell processes” (Jabbour-Zahab et al., 1992). There are conflicting reports of cilia within pearl sacs. Some reports indicate that cilia may be present in the early stages of pearl sac formation but not in later stages (Chatchavalvanich et al., 2010; Cochennec-Laureau et al., 2010), whereas others report variation in the presence or absence of cilia and the possible influence of the grafting process in this trait (Kishore and Southgate, 2015, 2016). Intriguingly, Dix (1973) reported that sacs producing nacreous pearls consist of a single, non-ciliated layer of epithelial cells, whereas a sac producing a “periostracal” (brown, organic layer) pearl consist of tall, ciliated epithelial cells. While no “periostracal” pearl sacs were investigated in this study, our findings are consistent with those of Dix (1973) and suggest a role for ciliation in nacre deposition. The association between cellular differentiation and the biomineralization of different calcium carbonate polymorphs has already been proposed for molluscs (Sud et al., 2002; Jolly et al., 2004; Jackson et al., 2006, 2007; McDougall et al., 2011; Marie et al., 2012) and bryozoans (Jacob et al., 2019).

### Underskin

**FIGURE 4:**
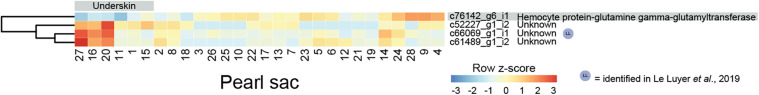
Heatmap of underskin-associated differentially expressed transcripts. Transcripts are displayed as horizontal rows and are clustered by similarity of expression profiles. Red indicates higher expression, and pearl sacs producing pearls with underskin are shown on the left of the plot, as indicated by the gray bar. One transcript was able to be annotated, indicated on the far right.

Four transcripts are significantly differentially expressed in pearl sacs that produced pearls with and without underskin (*n* = 6; Figure 4 and Supplementary Table S4). Only one of these transcripts, c52227_g1_i2, produced significant BLAST or Pfam hits, displaying similarity to arthropod hemocyte protein-glutamine gamma-glutamyltransferase. It is down-regulated in pearl sacs that yield pearls with underskin defects. Hemocyte protein-glutamine gamma-glutamyltransferase (transglutaminases) have been implicated in the immune response of the Pacific oyster *C. gigas* (Gueguen et al., 2003; Hart et al., 2016), suggesting that the underskin defect may be related to infection within the pearl sac. One of the unannotated transcripts (c66069_g1_i1) was likely also identified as being associated with pearl quality in the study by Le Luyer et al. (2019).

### Spots

In this study, only six pearls did not possess at least one “spot.” Despite the prevalence of this defect, no transcripts are significantly differentially expressed between pearl sacs producing pearls with and without spotting. We note that there is a large degree of variation associated with this defect, i.e., spots can either be raised or depressed, and either nacreous or opaque (Figure 1). Each type of spot may have a differing underlying cause, and it is possible that combining this defect into a single category has masked underlying gene expression differences.

### Pearl Weight

**FIGURE 5:**
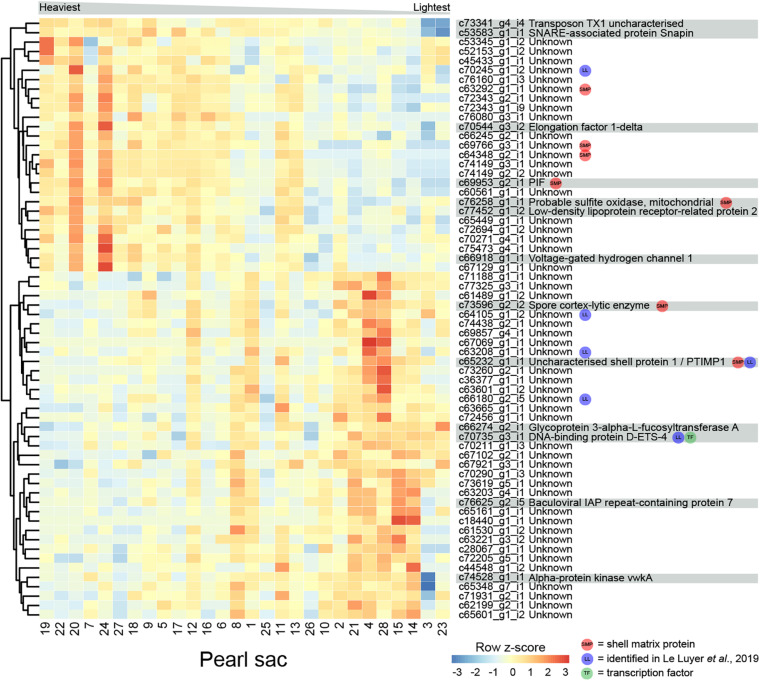
Heatmap of weight-associated differentially expressed transcripts. Transcripts are displayed as horizontal rows and are clustered by similarity of expression profiles. Red indicates higher expression, and pearl sacs producing heavier pearls are shown on the left of the plot, as indicated by the gray bar. Transcript annotations are indicated on the far right.

The weight of deposited pearl material in this study varied between 0.15 and 0.85 momme (0.56 and 3.19 g). 64 transcripts are significantly differentially expressed in relation to pearl weight (Figure 5 and Supplementary Table S5). Of these, 14 can be annotated. There are no functional categories over-represented within the differentially expressed genes.

Seven transcripts encode proteins with similarity to previously identified SMPs (Supplementary Table S5). c69953_g2_i1 encodes PIF, a protein that is cleaved into two components; Pif 80, an acidic protein that is involved in aragonite crystal formation, and Pif 97 that binds to chitin, in *P. fucata* (Suzuki et al., 2009, 2013). A second transcript encodes a homolog of “uncharacterized shell protein 1,” originally isolated from *P. margaritifera* shell (Joubert et al., 2010). Two other transcripts (c73596_g2_i2, annotated as “spore cortex-lytic enzyme,” and c76258_g1_i1, annotated as “probable sulfite oxidase,” mitochondrial) are similar to proteins isolated from *C. gigas* shell (Zhang et al., 2012), and the other three have similarity to uncharacterized SMPs. Six of the differentially expressed transcripts, including “uncharacterized shell protein 1,” were also likely identified by Le Luyer et al. (2019) (Supplementary Table S5).

One gene that was over-expressed in pearls with lower weights encodes an ETS4/PDEF transcription factor (Supplementary Figure S3). The ETS family of transcription factors play a wide range of roles in metazoans, including in neural development, vasculogenesis, hematopoiesis (Sharrocks, 2001; Yagi et al., 2003), and the regulation of spiculogenesis in sea urchins (Davidson et al., 2002). PDEF regulates the specification of secretory cells in vertebrates (Chen et al., 2009). It is possible that this transcription factor affects pearl development via the specification of particular biomineralization cell types.

Proteins predicted from other differentially expressed transcripts are similar to a range of proteins involved in general metabolism, including sulfite oxidase, voltage-gated hydrogen channel protein, and elongation factor 1-delta (Figure 5). These transcripts have higher expression in heavier pearls, possibly indicating overall higher metabolism in the corresponding pearl sacs.

### Multi-Character Differentially Expressed Transcripts

In total, 13 genes are significantly differentially expressed for more than one pearl characteristic (Table 3). Nine of the 43 genes that are differentially expressed between sacs producing pearls with high or low luster are also differentially expressed in association with calcification. Except for two genes, the expression levels of these multi-character transcripts correlate with high calcification and low luster pearls, suggesting an association between these traits. However, it is worth noting that pearls with high calcification are likely to be deemed to have a low luster, especially if the calcified proportion of the surface is high.

**TABLE 3:** Multi-trait differentially expressed transcripts.

Transcript	Annotation	Luster (low)	Calcification	Underskin	Weight (low)
c61489_g1_i2	–		↑	↑	↑
c71079_g1_i2	Lamin tail domain-containing protein 1	↓	↓		
c71079_g2_i1	–	↓	↓		
c71948_g1_i2	–	↓	↓		
c63138_g1_i3	Spondin-1	↓	↓		
c49420_g1_i1	–	↑	↓		
c53363_g1_i1	WAP four-disulfide core domain protein 18	↓	↓		
c67428_g1_i1	–	↑	↓		
c75828_g1_i1	Fibrocystin-L	↓	↓		
c72013_g3_i1	Acidic mammalian chitinase	↑	↑		
c101215_g1_i1	–	↓		↓	
c66069_g1_i1	–	↑		↑	
c63208_g1_i1	WAP domain containing			↑	↑

*Arrows indicate an up- and down-regulated gene expression in relation to each pearl quality trait.*

The other traits share very few differentially expressed genes, and no differentially expressed transcripts are shared between sacs producing pearls of differing luster and differing pearl weights. The lack of overlap in differentially expressed transcripts between all the pearl characteristics demonstrates that each is underpinned by unique transcriptional profiles.

Previous studies have investigated pearl sac gene expression in relation to pearl quality; however, in these studies, quality has generally been expressed as “high” or “low” without distinguishing between defect types. We expect that our “multi-character” genes are more likely to be uncovered by studies using a broader quality classification system. Three of the multi-character transcripts, c63208_g1_i1 (WAP-domain containing), c66069_g1_i1 (unannotated), and c72013_g3_i1 (chitinase), appear to have also been identified as quality-associated transcripts by Le Luyer et al. (2019). Notably, none of the well-studied SMPs that have previously been associated with pearl quality (e.g., MSI60, aspein, prismalin, or any shematrins) (Inoue et al., 2011a,b; Blay et al., 2018) are identified to be associated with any of the pearl quality characteristics investigated here.

## Conclusion

This study reveals that unique transcriptional profiles in pearl sacs underlie different pearl characteristics. These transcriptional profiles not only indicate possible causative mechanisms of particular pearl defects or undesirable traits, but also reveal hitherto unrecognized processes linked to biomineralization, for example, the potential role of ciliation and cytoskeletal elements. A number of known SMPs were differentially expressed in pearls displaying different traits, and further analysis of the role of these proteins will likely reveal their functional role across different shell polymorphs, i.e., in calcite or nacre, and how these are associated with particular pearl defects. The analysis of gene expression within sacs producing pearls with different characteristics also provides evidence for the involvement of the transcription factors FoxJ1 and ETS4 in biomineralization, providing candidates for the regulation of nacre formation and specification of biomineralization cell types in molluscs.

## Data Availability Statement

The datasets generated for this study can be found under NCBI BioProject PRJNA636870.

## Author Contributions

CM, PM, and BD conceived of the study. CM performed molecular work, bioinformatics and data analysis, and drafted the manuscript with contribution from all other authors. FA performed the transcriptome assembly and assisted with bioinformatic analysis. AS compiled the dataset of published SMP proteins and assisted with bioinformatic analysis. All authors have read and approved the final manuscript.

## Conflict of Interest

PM was employed by the company Clipper Pearls Pty Ltd. and Autore Pearls Pty Ltd. (Broome, WA, Australia) during the study. The remaining authors declare that the research was conducted in the absence of any commercial or financial relationships that could be construed as a potential conflict of interest.

## Footnotes

1 https://github.com/Trinotate/Trinotate.github.io/wiki

## Supplementary Material

Supplementary Figure 1 Photographs of pearls extracted for the pearl sacs used in this study (PDF).

Supplementary Figure 2 Maximum likelihood phylogenetic analysis of the *Pinctada maxima* FoxJ1 protein (PDF).

Supplementary Figure 3 Maximum likelihood phylogenetic analysis of the *Pinctada maxima* ETS4/PDEF protein (PDF).

Supplementary Table 1 Transcripts differentially expressed between sacs producing high vs low lustre pearls.

Supplementary Table 2 Transcripts differentially expressed between sacs producing pearls with ‘calcification’ and those without.

Supplementary Table 3 Gene Ontology ‘Biological Process’ enrichments within calcification-associated differentially expressed transcripts.

Supplementary Table 4 Transcripts differentially expressed between sacs producing pearls with ‘underskin’ and those without.

Supplementary Table 5 Transcripts differentially expressed between sacs producing pearls with different weights.
