# Supplementary material for: Pearl Sac Gene Expression Profiles Associated With Pearl Attributes in the Silver-Lip Pearl Oyster, Pinctada maxima
Source: Front Genet. 2021 Jan 8;11:597459. doi: 10.3389/fgene.2020.597459 (PMC7820862; doi:10.3389/fgene.2020.597459)
Supplement: Supplementary Figure 1 — Photographs of pearls extracted for the pearl sacs used in this study (PDF). [file Data_Sheet_1.PDF]

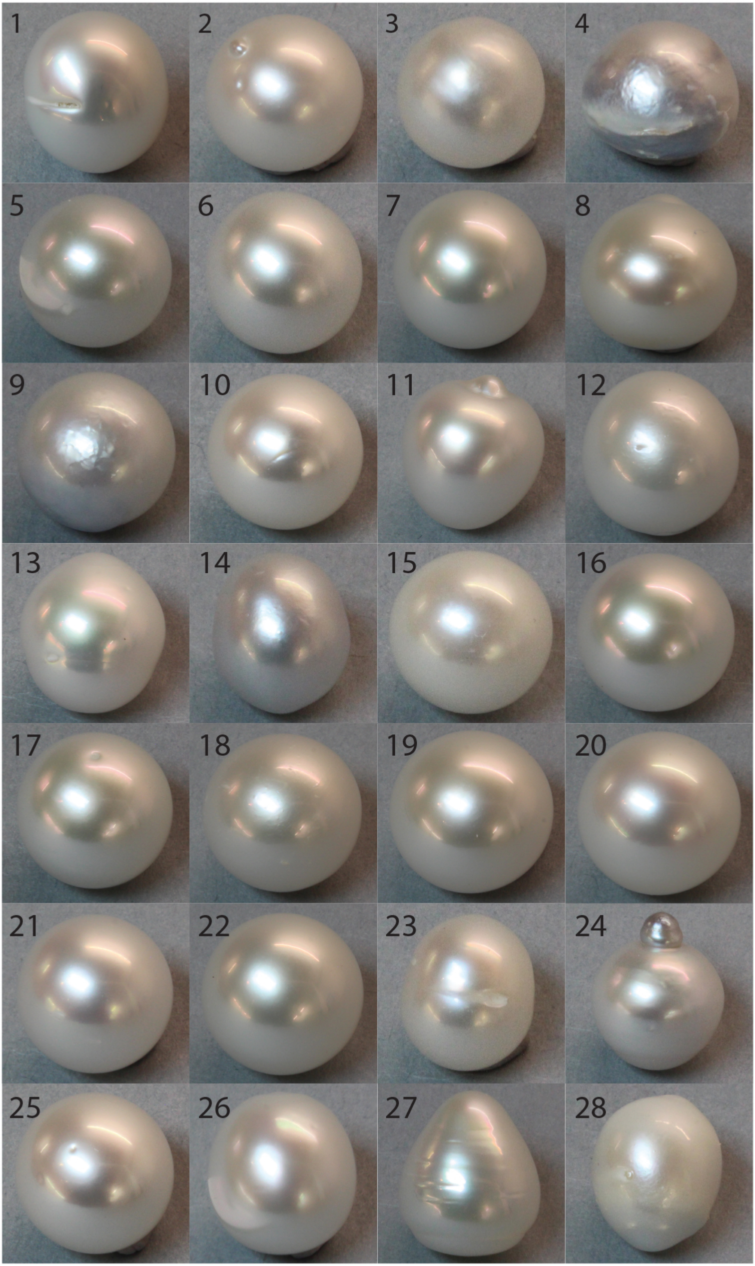

Figure S1. Photographs of pearls extracted from the pearl sacs used in this study. Pearl gradings can be found in Table 1.
