# Supplementary material for: Pearl Sac Gene Expression Profiles Associated With Pearl Attributes in the Silver-Lip Pearl Oyster, Pinctada maxima
Source: Front Genet. 2021 Jan 8;11:597459. doi: 10.3389/fgene.2020.597459 (PMC7820862; doi:10.3389/fgene.2020.597459)
Supplement: Supplementary Figure 2 — Maximum likelihood phylogenetic analysis of the Pinctada maxima FoxJ1 protein (PDF). [file Data_Sheet_2.PDF]

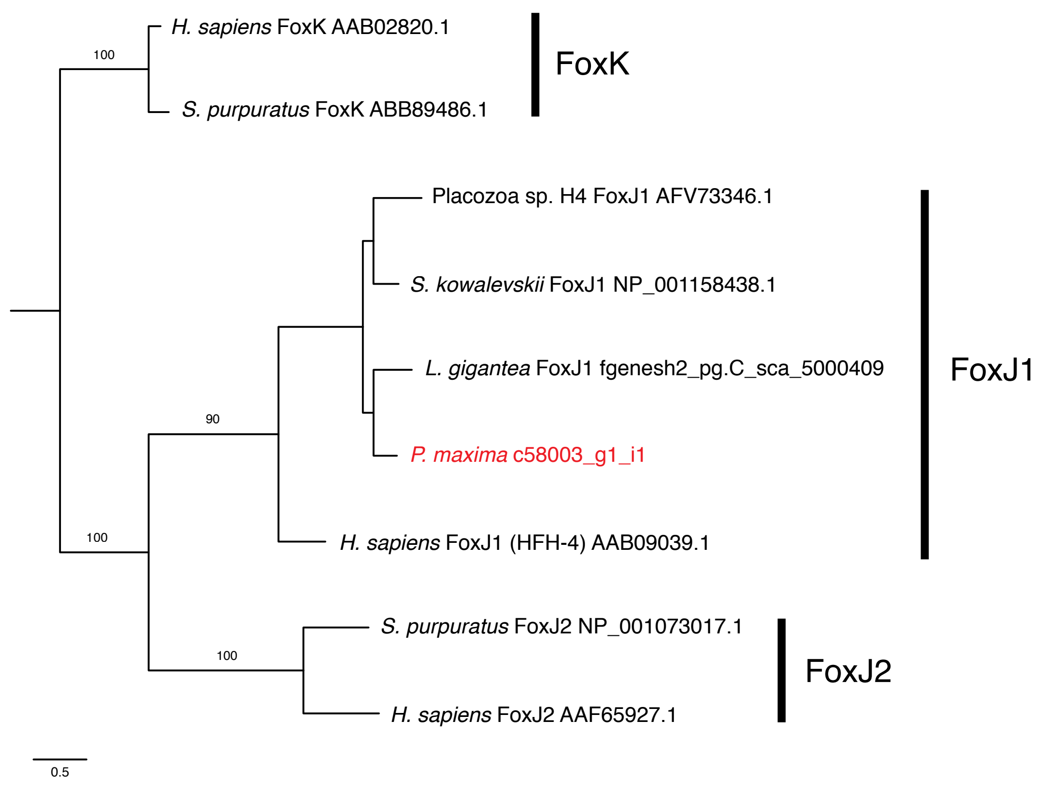

Figure S2. Maximum likelihood phylogenetic analysis of the *Pinctada maxima* FoxJ1 protein. Percent bootstrap values greater than 70 are displayed on the relevant branches. NCBI accession numbers are provided for each sequence, except for the *L. gigantea* sequence where the gene model number is provided (Simakov et al. 2013). The scale bar indicates the branch length for 0.5 amino acid substitutions.
