# Supplementary material for: Pearl Sac Gene Expression Profiles Associated With Pearl Attributes in the Silver-Lip Pearl Oyster, Pinctada maxima
Source: Front Genet. 2021 Jan 8;11:597459. doi: 10.3389/fgene.2020.597459 (PMC7820862; doi:10.3389/fgene.2020.597459)
Supplement: Supplementary Figure 3 — Maximum likelihood phylogenetic analysis of the Pinctada maxima ETS4/PDEF protein (PDF). [file Data_Sheet_3.PDF]

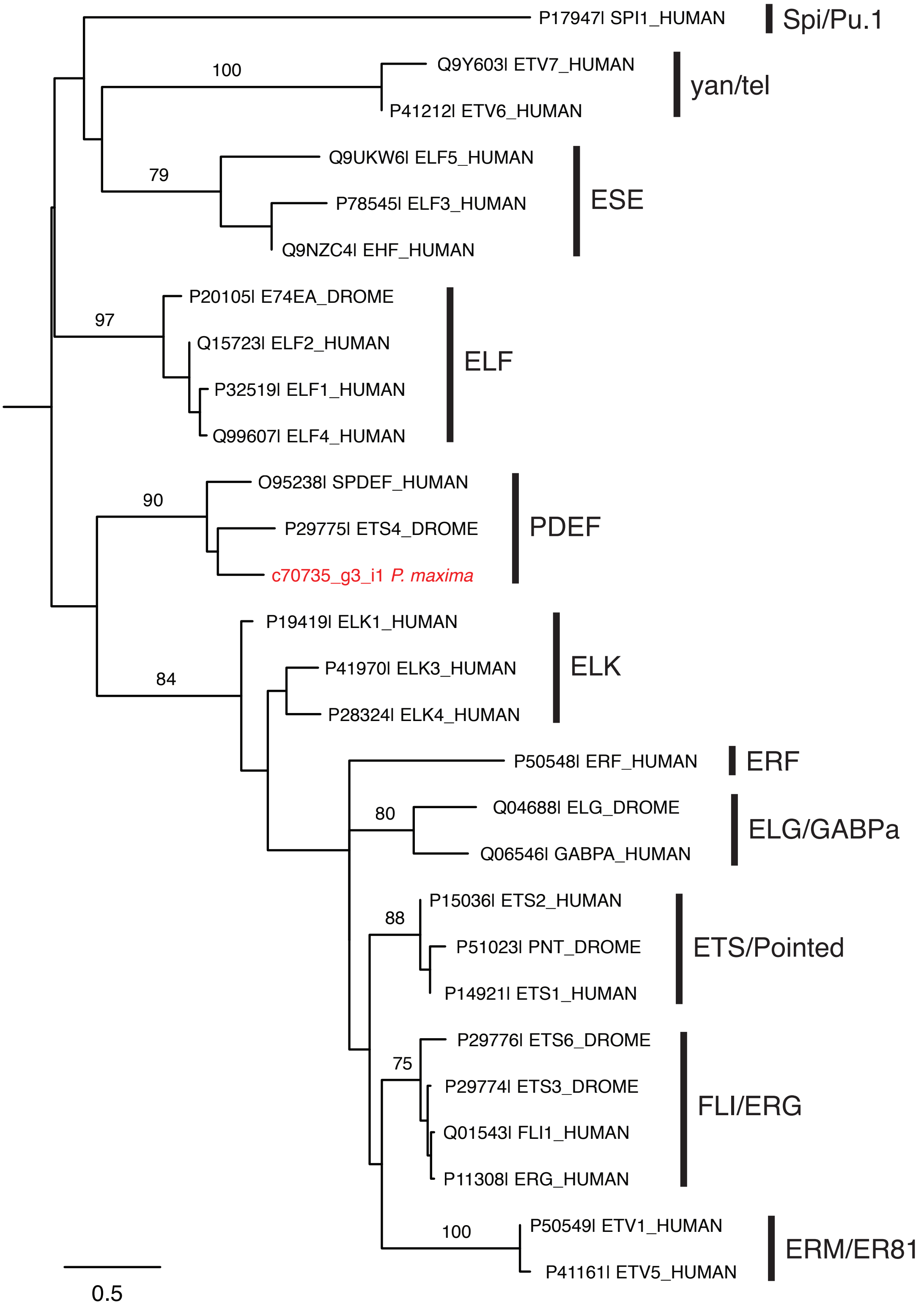

Figure S3. Maximum likelihood phylogenetic analysis of the *Pinctada maxima* ETS4/PDEF protein. Percent bootstrap values greater than 70 are displayed on the relevant branches. Analysis is based upon that in Yagi *et al* 2003, and Pfam accession numbers are provided for each sequence. The scale bar indicates the branch length for 0.5 amino acid substitutions. HUMAN; *Homo sapiens*, DROME; *Drosophila melanogaster.*
